# The Relationship of Affective Temperament and Emotional-Behavioral Difficulties to Internet Addiction in Turkish Teenagers

**DOI:** 10.1155/2013/961734

**Published:** 2013-03-28

**Authors:** Fatma Ozgun Ozturk, Mine Ekinci, Onder Ozturk, Fatih Canan

**Affiliations:** ^1^Department of Psychiatric Nursing, Faculty of Health Sciences, Ataturk University, 25240 Erzurum, Turkey; ^2^Department of Child and Adolescent Psychiatry, Ankara Children's Hospital, 06000 Ankara, Turkey; ^3^Psychiatry Clinic, Bolu Izzet Baysal Mental Health Hospital, 14030 Bolu, Turkey

## Abstract

The purpose of this study was to investigate the association of affective temperament profiles and emotional and behavioural characteristics with Internet addiction among high school students. The study sample included 303 high school students. A sociodemographic characteristics data form, internet addiction scale (IAS), the strengths and difficulties questionnaire, and the temperament evaluation of Memphis, Pisa, Paris, and San Diego autoquestionnaire were used to collect data. Of the sample, 6.6% were found to be addicted to Internet. Having a computer in the home (*P* < 0.001) and using the Internet for more than two years (*P* < 0.001) were found to be related to higher scores on the IAS. The prevalence rate of anxious temperament for Internet addicts was more than that for nonaddicts (*P* < 0.001). Dysthymic (*r* = 0.199; *P* < 0.01), cyclothymic (*r* = 0.249; *P* < 0.01), hyperthymic (*r* = 0.156; *P* < 0.01), irritable (*r* = 0.254; *P* < 0.01), and anxious (*r* = 0.205; *P* < 0.01) temperaments; conduct problems (*r* = 0.146; *P* < 0.05), hyperactivity-inattention (*r* = 0.133; *P* < 0.05), emotional symptoms (*r* = 0.138; *P* < 0.05), and total difficulties (*r* = 0.160; *P* < 0.01) were found to be correlated with IAS scores. According to these findings, there is a relation between the Internet addiction and affective temperament profiles, especially with anxious temperament. Furthermore, emotional and behavioural problems are more frequent in adolescents who have problematic Internet use.

## 1. Introduction

The Internet is a technology that facilitates accessing various kinds of information resources and information exchange easily via an inexpensive and safe way. Although a standardized definition of Internet addiction has not been uniformly agreed upon, some researchers define the Internet addiction as having less ability to control enthusiasm for Internet activities, losing the importance of the time without being connected to the Internet, extreme nervousness and aggressive behaviour when deprived, and progressive deterioration in work, and social and family functionings [1, 2]. Researchers point that the Internet addiction may be seen at every age in both sexes and begin at earlier ages than other addictions [3]. Prevalence statistics of Internet addiction among adolescents vary widely from 2% [4] to 20% [5] across cultures and societies. 

An Internet addict may typically spend 40–80 hours-weekly online [3]. For this reason, the Internet addiction may cause physical and social problems as well as psychological disturbances [6].

A number of studies have underlined the unfavorable effects of Internet addiction on physical and mental well-being and most of the adolescents with Internet addiction were also reported to have another psychiatric disorder [7, 8]. Mood disorders, substance use disorders, attention-deficit hyperactivity disorder (ADHD), disruptive behaviour disorders, anxiety disorders, sleep disorders, eating disorders, and epileptic seizures are some proven Internet addiction-related clinical situations [9].

Others have argued that the Internet addiction is actually a behaviour pattern which plays role in some of the negative cognitions that compensate for failed areas of life just as seen in depression [10]. In this context, excessive use of the Internet can be seen as a rewarding behavior, and through learning mechanisms, it may be used as an insufficient strategy to cope with some negative feelings [11]. 

Temperament traits of novelty or sensation seeking are reported to be significantly higher in substance users than in nonusers [12]. Most authors agree that these traits increase the risk of drug addiction in general [13], presumably because of an increased tendency to experiment with drugs. In studies investigating the temperament features of adolescents with Internet addiction, it was revealed that students with Internet addiction were easily affected by feeling, emotionally less stable, imaginative, absorbed in thought, self-sufficient, experimenting, and preferred their own decisions [7]. Adolescents with Internet addiction were also shown to have higher scores on neuroticism and psychoticism temperament categories than those of the control group [14]. However, to our knowledge, there is not a study in the literature addressing the correlation between affective temperament profiles and the Internet addiction.

The first aim of this study was to investigate Internet addiction and the relevance to the sociodemographic properties among a sample of Turkish adolescent population. Second, it was aimed to compare the affective temperament profiles and emotional and behavioural characteristics of adolescents with or without the Internet addiction.

## 2. Methods

### 2.1. Design and Sample

This is a descriptive and cross-sectional study. The study population included high school students attending Erzurum Ataturk High School in Turkey in the 2010-2011 academic year (*n* = 325). The study sample included 303 students who were present in classes on the day when data were collected, who agreed to participate in the study, and who filled in the questionnaires completely (response rate = 93.2%). 

### 2.2. Ethical Considerations

The ethical committee approval was obtained from the Institute of Health Sciences of Ataturk University. Approval was obtained from the director of Erzurum Ataturk High School. The students who were given information about the study and who accepted to participate in the study were included. Also, approval was obtained from the Directorate of School Education, affiliating with the Ministry of Education.

### 2.3. Data Collection

Four instruments were used to collect data: a sociodemographic characteristics data form, internet addiction scale, the strengths and difficulties questionnaire, and the temperament evaluation of Memphis, Pisa, Paris, and San Diego autoquestionnaire. The students provided their responses while in a counseling course class. Completion of the instruments took an average of 40 minutes. 

### 2.4. Data Gathering Tools

#### 2.4.1. Sociodemographic Characteristics Data Form

We developed a 12-item sociodemographic questionnaire with items pertaining to age, sex, grade, average monthly household income, extent and type of internet use (e.g., “Where do you use the Internet?”), and presence of the computer in the home.

#### 2.4.2. Internet Addiction Scale (IAS)

The IAS [15] is a self-report instrument consisting of 31 items (e.g., “I have stayed on the Internet longer than I intended to,” “I feel that life without the Internet would be boring and empty,” “I have attempted to spend less time on the Internet but I have been unable to do so.”) based on the Diagnostic and Statistical Manual of Mental Disorders, Fourth Edition, substance dependence criteria, and 2 additional criteria recommended by Griffiths [16]. The IAS is a highly reliable and internally consistent measure (Cronbach *α* = .95). The scale was translated into Turkish, and psychometric properties of the Turkish version of the scale were evaluated among high school students revealing a highly significant test-retest reliability [17]. An interitem reliability reduced the initial scale from 31 to 27 items (with Cronbach *α* of .94). Scale items are rated on a 5-point Likert scale (1, never; 2, rarely; 3, sometimes; 4, frequently; 5, always), with higher scores representing greater Internet addiction. A cutoff score of 81 (3 × 27 items) was suggested as indicative of Internet addiction. 

#### 2.4.3. The Strengths and Difficulties Questionnaire (SDQ)

The SDQ [18] was developed to determine adolescents' areas of strengths and problematic behaviours. The tool contains 25 questions which asks about behavioural characteristics, some of which are positive, and some of which are negative. These questions are listed under five subheadings: (1) conduct problems; (2) hyperactivity-inattention; (3) emotional symptoms; (4) peer problems; and (5) prosocial behaviour. The first four subheadings are categorized under “total difficulty score.” This score varies between 0 and 40. The validity and reliability of the Turkish version of SDQ was performed by Güvenir et al. [19] with an acceptable internal consistency (Cronbach's alpha = 0.73).

#### 2.4.4. The Temperament Evaluation of Memphis, Pisa, Paris, and San Diego Autoquestionnaire (TEMPS-A)

The autoquestionnaire version of the temperament evaluation of Memphis, Pisa, Paris, and San Diego (TEMPS-A) is a self-report instrument developed by Akiskal et al. [20]. It has been validated for use in both psychiatrically ill and healthy individuals. The complete questionnaire measures affective temperamental traits, present in the subject's whole life, represented in five dimensional scales: depressive, cyclothymic, hyperthymic, irritable, and anxious. In this study, the Turkish version was used [21]. 

### 2.5. Data Analyses

Statistical Package for Social Sciences software (SPSS 15, Chicago, IL, USA) was used for the analysis. Descriptive parameters were shown as mean ± standard deviation or in percentages. Continuous variables were compared using the Student *t* test. Pearson's chi-square test was used to analyze the differences in means and proportions between groups. Spearman's or Pearson's correlation tests were used to evaluate the association between the IAS and the subscales of the SDQ and the TEMPS-A. A *P* value of <0.05 was considered significant. 

## 3. Results

A total of 210 boys (69.2%) and 92 girls (30.8%) completed the scale and questionnaires. Of the sample, 20 (6.6%) were found to be addicted to Internet according to the IAS. The proportion of boys who were classified as Internet addicts was 6.2%. For girls, the corresponding proportion was 7.6%; the difference was not statistically significant. Having a computer in the home was found to be significantly related with Internet addiction.  Table 1 lists the baseline subject characteristics by the presence or absence of Internet addiction.

The mean IAS scores were significantly higher in adolescents who had a computer in home than those who had not (*P* < 0.001). Additionally, students who had been using Internet for more than two years were found to score higher on the IAS than those who had been using Internet for two years or less (*P* < 0.001). The IAS scores were also significantly higher in adolescents who had been using the Internet in home than in those who had been using the Internet in other places (*P* < 0.001). 

The prevalence rate of anxious temperament for Internet addicts was 15%, whereas that for nonaddicts, it was 2.8% (*P* < 0.001). Temperament subtypes and their distribution in terms of the Internet addiction status are shown in Table 2. The mean IAS scores were found to be higher in adolescents with anxious temperament (63.9 ± 25.3) than those without anxious temperament (47.9 ± 18.1) (*P* < 0.05). Presence or absence of other temperament subtypes was not associated with significantly different scores on the IAS. According to Pearson's correlation coefficient, significant correlations were detected between Internet addiction and dysthymic (*r* = 0.199; *P* < 0.01), cyclothymic (*r* = 0.249; *P* < 0.01), hyperthymic (*r* = 0.156; *P* < 0.01), irritable (*r* = 0.254; *P* < 0.01), and anxious (*r* = 0.205; *P* < 0.01) temperaments.

Adolescents with and without Internet addiction were also compared according to their TEMPS-A and SDQ scores (Table 3). Although no difference was observed in TEMPS-A scores, students with Internet addiction scored higher on conduct problems (*P* < 0.05) and total difficulties (*P* < 0.05) subscales of SDQ than students without Internet addiction. Moreover, there was a positive and statistically significant correlation between IAS and conduct problems (*r* = 0.146; *P* < 0.05), hyperactivity-inattention (*r* = 0.133; *P* < 0.05), emotional symptoms (*r* = 0.138; *P* < 0.05), and total difficulties (*r* = 0.160; *P* < 0.01).

## 4. Discussion

In the present study, the prevalence of Internet addiction was found to be 6.6%, which is similar with the rate found in other studies evaluating similarly aged students [22, 23]. According to our findings, the risk of becoming an Internet addict increases with the increase in the accesibility of Internet. Additionally, Internet use with a duration of more than two years was also found to be related to increased Internet addiction risk.

In our study, presumably because of low participation rates in girls, there was no significant difference between boys and girls according to IAS scores. Contrary to our finding, Turkey Statistical Institute has stated that computer and Internet use was more prevalent among boys than in girls in 2010 data [24]. Other studies from Turkey have also shown that boys were more prone to the effects of harmful Internet use [17, 25]. 

In a study which evaluated 535 primary school students using Child Behavior Checklists, ADHD scores have been found to be higher in adolescents with Internet addiction than in those without [26]. Additionally, Yen et al. [27], evaluating 2793 college students, revealed that there was a relationship between the Internet addiction and attention deficit hyperactivity disorder (ADHD). They have also shown that the most prominent relation between the Internet addiction was with attention deficit symptom cluster. Similarly, in the present study, Internet addiction scores were found to be positively related to attention deficit and hyperactivity scores. According to the “rewarding withdrawal syndrome,” because of the D2 receptor deficiency, children with ADHD have marked tendency to predisposition for pathological gambling, substance and alcohol use, and impulsive and compulsive behaviours [28]. The Internet addiction, according to the “reward deficiency hypothesis,” may function as an “unnatural reward” and may accompany ADHD symptoms by this way [26].

Dependent personality traits have been shown to be related with impulsivity, novelty seeking, psychoticism, and social relation problems in several studies [29, 30]. Landers and Lounsbury [31] evaluated 117 undergraduate students and found that the Internet usage was negatively related to three of the big five traits, agreeableness, conscientiousness, and extraversion as well as two narrow traits; optimism and work drive, and positively related to tough-mindedness. In a study conducted among college students in Turkey, psychoticism was shown to be the only personality dimension related to establishing new relationships and having “Internet only” friends. Moreover, extroversion was the only personality dimension that is related to maintaining long-distance relationships and supporting daily face-to-face relationships [32]. In our study, a positive and highly significant correlation was found between the Internet addiction scores and depressive, cyclothymic, hyperthymic, irritable, and anxious temperament scores. Furthermore, the frequency of anxious temperament was found to be significantly higher in students with Internet addiction than those without. 

Behavioural addictions demonstrate the core features of physical and psychological addictions such as mental rumination, mood variability, tolerance, withdrawal, interpersonal conflict, and relapse [33]. According to the “self-medication hypothesis,” the patients usually use the substances to change their unwanted temperament status, to reduce their unbearable anxiety, and to cope with cognitive impairments [34]. This can be seen in the Internet addiction, which is also a behavioral addiction. Namely, repeating efforts to get online may be a way of decreasing the severity of withdrawal symptoms such as anxiety. Additionally, the explanation of increased Internet addiction frequency in individuals with anxious temperament may be related to “self-medication hypothesis.”

Adolescents who are deprived of emotional and psychological support have been reported to be under increased risk for Internet addiction [35]. Morahan-Martin and Schumacher [36] revealed that 22.7% of the Internet users had trouble with peer and family relations and had difficulty in work and school activities because of the Internet usage. In our study sample, total strength scores and conduct problems scores of the SDQ have been found to be significantly higher in the students with Internet addiction. Also, there was a positive correlation between the Internet addiction scores and total difficulties, conduct problems, hyperactivity-inattention, and emotional symptoms scores. According to these findings, there is an association between problematic Internet use and emotional and behavioural problems.

*Limitations. *There are several limitations of the present study. First, since the sample of this study included students of a high school, the results of the study cannot be generalized to the larger population in Turkey. Second, the sample size was modest to draw definite conclusions. Also, high school education was not mandatory in Turkey when this study was conducted. Families in the East and Southeast of Turkey invest more in the education of their sons than their daughters [37]. Thus, our study populations comprise 69.2% boys and 30.8% girls. Finally, the cross-sectional research design of the present study could not confirm causal relationships of temperament profiles and behavioural problems with Internet addiction.

## 5. Conclusions

According to the findings of the present study, Internet addiction is a relatively common phenomena among the adolescents. There is a relation between the Internet addiction and attention deficit and hyperactivity symptoms and also with anxious temperament. Furthermore, behavioural problems are more frequent in adolescents who have problematic Internet use. Because of the cross-sectional nature of this study, it is not possible to define the direction of the causality of the results. There is a need for further prospective studies evaluating the temperament features of adolescents who are at risk for the Internet addiction in larger study populations.

## Figures and Tables

**Table 1:** Sociodemographic properties of adolescents in respect to Internet addiction status (chi-square test).

	Nonaddicted (*n* = 283)	Addicted (*n* = 20)	*P* value
Gender			
Girls (*n* = 93)	86 (92.4%)	7 (7.6%)	0.66
Boys (*n* = 210)	197 (93.8%)	13 (6.2%)	
Age			
14 years (*n* = 46)	44 (95.7%)	2 (4.3%)	0.38
15 years (*n* = 117)	112 (95.7%)	5 (4.3%)	
16 years (*n* = 89)	81 (91%)	8 (9%)	
17 years (*n* = 51)	46 (90.2%)	5 (9.8%)	
Grade			
9 (*n* = 173)	164 (94.8%)	9 (5.2%)	0.48
10 (*n* = 89)	82 (92.1%)	7 (7.9%)	
11 (*n* = 41)	37 (90.2%)	4 (9.8%)	
Residence			
Town (*n* = 53)	51 (96.2%)	2 (3.8%)	0.28
City center (*n* = 250)	232 (92.8%)	18 (7.2%)	
Household income			
<1000 TL (n = 188)	179 (95.2%)	9 (4.8%)	0.08
≥1000 TL (n = 115)	104 (90.4%)	11 (9.6%)	
Computer in the home			
Yes (n = 162)	146 (90.1%)	16 (9.9%)	**0.01**
No (n = 141)	137 (97.2%)	4 (2.8%)	
Internet usage time			
<1 year (n = 85)	81 (95.3%)	4 (4.7%)	0.06
1-2 years (n = 95)	92 (96.8%)	3 (3.2%)	
>2 years (n = 123)	110 (89.4%)	13 (10.6%)	
Place of internet use			
Internet cafe (*n* = 152)	147 (96.7%)	5 (3.3%)	0.13
House (*n* = 119)	107 (89.9%)	12 (10.1%)	
School (*n* = 13)	12 (92.3%)	1 (7.7%)	
Others (*n* = 19)	17 (89.5%)	2 (10.5%)	
Control of internet use			
Himself/herself (*n* = 192)	180 (93.7%)	12 (6.3%)	0.91
Mother (*n* = 50)	47 (94%)	3 (6%)	
Father (*n* = 44)	40 (91.9%)	4 (9.1%)	
Others (*n* = 17)	16 (94.1%)	1 (5.9%)	

TL: Turkish lira.

**Table 2:** Temperament characteristics of adolescents in respect to Internet addiction status.

	Nonaddicted	Addicted	P value
Dysthymic			
Yes (*n* = 5)	4 (80%)	1 (20%)	0.291
No (*n* = 298)	279 (93.2%)	19 (6.8%)	
Cyclothymic			
Yes (*n* = 1)	1 (100%)	0 (0%)	0.934
No (*n* = 302)	282 (93.4%)	20 (6.6%)	
Hyperthymic			
Yes (*n* = 2)	2 (100%)	0 (0%)	0.872
No (*n* = 301)	281 (92.9%)	20 (7.1%)	
Irritable			
Yes (*n* = 13)	13 (100%)	0 (0%)	0.404
No (*n* = 290)	270 (93.1%)	20 (6.9%)	
Anxious			
Yes (*n* = 11)	8 (72.8%)	3 (27.2%)	**0.029**
No (*n* = 292)	275 (94.2%)	17 (5.8%)	

**Table 3:** Comparison of TEMPS-A and SDQ mean scores of students with and without internet addiction.

	Nonaddicted (mean ± SD)	Addicted (mean ± SD)	*P* value
TEMPS-A			
Dysthymic	5.9 ± 2.8	6.3 ± 2.9	0.64
Cyclothymic	8.6 ± 4.1	9.9 ± 3.2	0.14
Hyperthymic	11.5 ± 3.8	12.9 ± 3.2	0.08
Irritable	4.9 ± 4.0	6.0 ± 3.4	0.13
Anxious	6.0 ± 4.6	8.3 ± 6.4	0.13
SDQ			
Conduct problems	2.6 ± 1.8	3.9 ± 2.3	**0.02**
Hyperactivity-inattention	3.8 ± 1.9	4.5 ± 1.8	0.10
Emotional symptoms	2.7 ± 2.4	3.7 ± 2.5	0.09
Peer problems	3.6 ± 1.8	3.9 ± 2.1	0.77
Prosocial behaviour	7.3 ± 2.5	7.8 ± 2.0	0.58
Total difficulty score	12.8 ± 5.7	16.1 ± 6.3	**0.02**

SDQ: the strengths and difficulties questionnaire (SDQ); TEMPS-A: the temperament evaluation of Memphis, Pisa, Paris, and San Diego Autoquestionnaire.

## References

[1] Young KS (1996). Psychology of computer use: XL. Addictive use of the Internet: a case that breaks the stereotype. *Psychological Reports*.

[2] Young KS (1998). Internet addiction: the emergence of a new clinical disorder. *CyberPsychology & Behavior*.

[3] Whang LSM, Lee S, Chang G (2003). Internet over-users’ psychological profiles: a behavior sampling analysis on Internet addiction. *Cyberpsychology and Behavior*.

[4] Johansson A, Götestam KG (2004). Internet addiction: characteristics of a questionnaire and prevalence in Norwegian youth (12–18 years). *Scandinavian Journal of Psychology*.

[5] Ha JH, Yoo HJ, Cho IH, Chin B, Shin D, Kim JH (2006). Psychiatric comorbidity assessed in Korean children and adolescents who screen positive for internet addiction. *Journal of Clinical Psychiatry*.

[6] Hur MH (2006). Demographic, habitual, and socioeconomic determinants of Internet addiction disorder: an empirical study of Korean teenagers. *Cyberpsychology and Behavior*.

[7] Yang CK, Choe BM, Baity M, Lee JH, Cho JS (2005). SCL-90-R and 16PF profiles of senior high school students with excessive internet use. *Canadian Journal of Psychiatry*.

[8] Yen JY, Ko CH, Yen CF, Chen SH, Chung WL, Chen CC (2008). Psychiatric symptoms in adolescents with Internet addiction: comparison with substance use. *Psychiatry and Clinical Neurosciences*.

[9] Ko C-H, Yen J-Y, Yen C-F, Chen C-S (2012). The association between Internet addiction and psychiatric disorder: a review of the literature. *European Psychiatry*.

[10] Davis RA (2001). Cognitive-behavioral model of pathological Internet use. *Computers in Human Behavior*.

[11] Du YS, Jiang W, Vance A (2010). Longer term effect of randomized, controlled group cognitive behavioural therapy for Internet addiction in adolescent students in Shanghai. *Australian and New Zealand Journal of Psychiatry*.

[12] Mâsse LC, Tremblay RE (1997). Behavior of boys in kindergarten and the onset of substance use during adolescence. *Archives of General Psychiatry*.

[13] Le Bon O, Basiaux P, Streel E (2004). Personality profile and drug of choice; A multivariate analysis using Cloninger’s TCI on heroin addicts, alcoholics, and a random population group. *Drug and Alcohol Dependence*.

[14] Cao F, Su L (2007). Internet addiction among Chinese adolescents: prevalence and psychological features. *Child: Care, Health and Development*.

[15] Nichols LA, Nicki R (2004). Development of a psychometrically sound internet addiction scale: a preliminary step. *Psychology of Addictive Behaviors*.

[16] Griffiths M, Gackenbach J (1998). Internet addiction: does it really exist?. *Psychology and the Internet*.

[17] Canan F, Ataoglu A, Nichols LA, Yildirim T, Ozturk O (2010). Evaluation of psychometric properties of the internet addiction scale in a sample of Turkish high school students. *Cyberpsychology, Behavior, and Social Networking*.

[18] Goodman R (1999). The extended version of the Strengths and Difficulties Questionnaire as a guide to child psychiatric caseness and consequent burden. *Journal of Child Psychology and Psychiatry and Allied Disciplines*.

[19] Güvenir T, Özbek A, Baykara B, Şentürk B, İncekaş S Validation and reliability study of The Strengths and Difficulties Questionnaire (SDQ).

[20] Akiskal HS, Akiskal KK, Haykal RF, Manning JS, Connor PD (2005). TEMPS-A: progress towards validation of a self-rated clinical version of the Temperament Evaluation of the Memphis, Pisa, Paris, and San Diego Autoquestionnaire. *Journal of Affective Disorders*.

[21] Vahip S, Kesebir S, Alkan M, Yazici O, Akiskal KK, Akiskal HS (2005). Affective temperaments in clinically-well subjects in Turkey: initial psychometric data on the TEMPS-A. *Journal of Affective Disorders*.

[22] Park SK, Kim JY, Cho CB (2008). Prevalence of Internet addiction and correlations with family factors among South Korean adolescents. *Adolescence*.

[23] Lin SSJ, Tsai CC (2002). Sensation seeking and internet dependence of Taiwanese high school adolescents. *Computers in Human Behavior*.

[24] Turkish Statistical Institute http://www.turkstat.gov.tr.

[25] Canan F, Ataoglu A, Ozcetin A, Icmeli C (2012). The association between Internet addiction and dissociation among Turkish college students. *Comprehensive Psychiatry*.

[26] Hee JY, Soo CC, Ha J (2004). Attention deficit hyperactivity symptoms and Internet addiction. *Psychiatry and Clinical Neurosciences*.

[27] Yen JY, Yen CF, Chen CS, Tang TC, Ko CH (2009). The association between adult ADHD symptoms and internet addiction among college students: the gender difference. *Cyberpsychology and Behavior*.

[28] Blum K, Braverman ER, Holder JM (2000). Reward deficiency syndrome: a biogenetic model for the diagnosis and treatment of impulsive, addictive, and compulsive behaviors. *Journal of Psychoactive Drugs*.

[29] Allen TJ, Moeller FG, Rhoades HM, Cherek DR (1998). Impulsivity and history of drug dependence. *Drug and Alcohol Dependence*.

[30] Eysenck HJ (1997). Addiction, personality and motivation. *Human Psychopharmacology*.

[31] Landers RN, Lounsbury JW (2006). An investigation of Big Five and narrow personality traits in relation to Internet usage. *Computers in Human Behavior*.

[32] Tosun LP, Lajunen T (2010). Does Internet use reflect your personality? Relationship between Eysenck’s personality dimensions and Internet use. *Computers in Human Behavior*.

[33] Donovan JE (2004). Adolescent alcohol initiation: a review of psychosocial risk factors. *Journal of Adolescent Health*.

[34] Mirin SM, Weiss RD, Michael J, Griffin ML (1988). Psychopathology in substance abusers: diagnosis and treatment. *American Journal of Drug and Alcohol Abuse*.

[35] Durkee T, Kaess M, Carli V (2012). Prevalence of pathological internet use among adolescents in Europe: demographic and social factors. *Addiction*.

[36] Morahan-Martin J, Schumacher P (2000). Incidence and correlates of pathological internet use among college students. *Computers in Human Behavior*.

[37] O’Dwyer J, Aksit N, Sands M (2010). Expanding educational access in Eastern Turkey: a new initiative. *International Journal of Educational Development*.

